# Structural Crack Detection Using DPP-BOTDA and Crack-Induced Features of the Brillouin Gain Spectrum

**DOI:** 10.3390/s20236947

**Published:** 2020-12-04

**Authors:** Dongyu Zhang, Yang Yang, Jinlong Xu, Li Ni, Hui Li

**Affiliations:** 1 Key Lab of Structures Dynamic Behavior and Control, Ministry of Education, Harbin Institute of Technology, Harbin 150090, China; 17S133146@hit.edu.cn (Y.Y.); nili@hit.edu.cn (L.N.); lihui@hit.edu.cn (H.L.); 2 School of Civil Engineering, Harbin Institute of Technology, Harbin 150090, China; 3 Hongfeng Control Technology Co., Ltd. of Hubei Sanjiang Aerospace Group, Xiaogan 432100, China; jinlongxu@hit.edu.cn

**Keywords:** DPP-BOTDA, Brillouin gain spectrum, distributed strain sensing, structural crack detection

## Abstract

Structural damage generally initiates in the form of structural cracks. Thus, developing efficient crack detection techniques is of great importance for the structural health monitoring. In this paper, a new crack identification method is proposed, which is based on the differential pulse-width pair Brillouin optical time domain analysis (DPP-BOTDA) technology and the irregular features of Brillouin gain spectrum (BGS) in the fiber due to structural cracks. The proposed method provides a new way to detect and quantify structural cracks without knowing the strain in the structure. First, the working mechanism of DPP-BOTDA is introduced to illustrate the reason that the DPP-BOTDA, compared to traditional BOTDA technique, can significantly improve the spatial resolution of distributed strain sensing, which is critical for structural crack detection. Then, the BGSs in the fiber with the presence of structural cracks, measured by the DPP-BOTDA, are numerically simulated, from which the crack-induced irregular features of the BGS are summarized. Based these irregular features, new structural crack detection and quantification methods are proposed, which are found to be independent of structural stain. Finally, an experiment is conducted on a simple supported reinforced concrete (RC) beam. The results demonstrate that by using the BGS measured by the DPP-BOTDA, the proposed structural crack identification method successfully detects the occurrence of structural cracks and relatively accurately predicts the crack widths.

## 1. Introduction

Important civil structures, such as dams, bridges, buildings, etc., play crucial roles in supporting normal operation of human society. However, with gradual deterioration of construction materials and environmental corrosion, the structures will be unavoidably damaged. The damages generally initiate in the form of structural cracks, which allow external corrosive materials (e.g., chloride ion) to penetrate deeper inside the structure, accelerating the structural damage progress. Therefore, developing efficient techniques that can monitor the initiation and evolution of cracks on the surface of and inside structures will be of great value to accurately access structural damage condition and ensure the safe operation of the structure.

Traditional structural crack detection mostly relies on human vision inspection, which is time and labor consuming. Some nondestructive test (NDT) methods, such as piezoelectricity transducer (PZT) sensors [1,2] and eddy current-based technologies [3] etc., are also effective to detect structural cracks given that the proximate positions of potential cracks are known as a priori. Recently, with fast development of (unmanned aerial vehicle) UAV and computer vision recognition technologies [4], the surface images of large civil structures can be more easily collected and be more efficiently processed to quickly detect structural cracks. However, most of these techniques can only find the structural surface cracks and cannot detect the structural internal cracks. Moreover, they cannot continuously monitor the evolution of cracks either, which is critical to determine structural safety. In order to obtain the evolution information of structural cracks, some strain gauges or displacement transducers are needed, which, however, can only monitor structural cracks at limited discrete locations.

In the late of the last century, the Brillouin optical time-domain reflectometry/analysis (BOTDR/A) technologies [5,6,7] have been emerged as promising methods for distributed structural health monitoring. Different from the point-type sensors, like strain gauges, that can only measure the structural strain at limited discrete locations, the BOTDR/A sensing system uses a standard single-mode optical fiber as both sensing and signal transmission medium to measure structural strains along the fiber; thus, the BOTDR/A become inexpensive and efficient solutions to obtaining structural distributed strain and possibly detecting structural damage (e.g., cracks) along the fiber. Compared to the BOTDR, the BOTDA takes advantage of the stimulated Brillouin scattering (SBS) effect to significantly improve the signal-to-noise ratio (SNR), which greatly enhances its sensing accuracy and distance. So far, the longest sensing distance of the BOTDA reported is 150 km [8], which makes the BOTDA very suitable for distributed strain sensing of very large civil structures, like tunnels and long-span bridges.

Due to the promising application potential of BOTDR/A technology in structural health monitoring, many scholars have conducted the research on using them to detect structural damage [9,10,11,12,13]. Goldfeld et al. [10] proposed several crack indicators that correlate the BOTDR/A strain readings to the crack number on a RC beams to infer the structural damage status. Feng et al. [11] utilized a shear lag model to study the fiber strain distribution near structural cracking, considering the protective coating material of the fiber to be in both elastic and elasto-plastic stages. Deif et al. [12] utilized a multi-peak fitting method to detect the abnormal strain readings due to structural cracks. Liu et al. [13] utilized the BOTDA to obtain the strains of the bridge superstructure, which was applied to bridge damage localization using quasi-static strain influence lines. Li et al. [14] combined the BOTDA with long-gauge fiber grating sensors on a single fiber to simultaneously monitor the multiple parameters related to the health condition of slopes.

In spite of the advance of many researches, accurately detecting minor structural damage, like cracks, using BOTDR/A still remains challenging, which is primarily due to limited spatial resolution of strain sensing of these technologies. The strain spatial resolution of BOTDR/A is generally larger than 1 m, but the width of structural cracks is generally on the order of one-tenth millimeters. Although the cracks may lead to large strains in the fiber near their vicinity, their effects are diluted in the BOTDR/A strain measurements due to the BOTDR/A’s limited spatial resolution. Therefore, improving the spatial resolution become an important issue to enhance BOTDA capability to detect structural micro cracks. To cope with this problem, several novel techniques have been proposed. One of them is pre-pump pulse BOTDA (PPP-BOTDA), which utilizes a pre-pump pulse to stimulate enough phonons for Brillouin scattering effect before arrival of a much shorter pump signal to conduct strain measurement, achieving centimeter level of spatial resolution [15]. Several researchers have verified theoretically and experimentally the feasibility of using the PPP-BOTDA to detect structural surface cracks [16,17]. However, the sensing distance of the PPP-BOTDA is limited by the power depletion of pre-pump pulse; and because of the influence of the pre-pump pulse on the short sensing pulse, the PPP-BOTDA may not accurately locate the position of structural cracks [17]. Another technology to improve the spatial resolution of BOTDA is differential pulse-width pair BOTDA (DPP-BOTDA), which is implemented by separately using two pump pulse lights with slightly different pulse widths; and the differential Brillouin gain of the two pump pulse lights is utilized to estimate the structural strain. It was shown that the spatial resolution of the DPP-BOTDA is determined by the difference of the two pulse widths. Since it is much easier to tune the width difference of the two pump pulses than to shorten the width of single pump pulse, the DPP-BOTDA provide a new feasible way of greatly enhancing the spatial resolution of strain sensing. Xu et al. [18] made use of DPP-BOTDA to detect the blade structural fatigue damage of wind turbine. But, to the best of authors’ knowledge, few studies have been conducted to utilize the DPP-BOTDA to detect the structural cracks.

In this paper, a systematic research is conducted on using the DPP-BOTDA to detect and quantify the structural cracks. The paper is organized as follows. First, the working mechanism of DPP-BOTDA is introduced to explain the reason that the DPP-BOTDA can significantly improve the spatial resolution of distributed strain sensing. Then, the BGSs in the fiber with the presence of structural cracks are numerically simulated, from which the crack-induced irregular features of the BGS are thoroughly investigated. Based these irregular features, new structural crack detection and quantification methods are proposed, which are proved to be independent of structural stain. Finally, an experiment is conducted on a simple supported reinforced concrete (RC) beam. The results demonstrate that by using the BGS measured by the DPP-BOTDA, the proposed structural crack identification method successfully detects the crack locations and relatively accurately predicts the crack widths.

## 2. Working Mechanism of DPP-BOTDA

In this section, the working mechanism of DPP-BOTDA and the theoretical model to calculate the BGS of the fiber are first reviewed. Then, the method to simulate the BGS of the fiber with non-uniformly distributed strain is derived, which will be used thereafter to compute the BGS of the fiber that is subject to structural cracks in Section 3.

### 2.1. Working Mechanism of DPP-BOTDA Based Distributed Strain and Temperature Sensing

In the BOTDA system, one pump pulse light and one continuous wave (CW) probe light are inject into the two ends of the fiber respectively and counter-propagate to each other. The pump pulse light interacts with the CW probe light to induce the stimulated Brillouin scattering (SBS) effect, in which some energy of the pump pulse light is transferred to the CW probe light. This energy transition, known as Brillouin gain, are measured by the BODTA demodulator at the starting end of the fiber. Then, via tuning the frequency difference of the pump pulse and CW probe lights, the whole Brillouin gain spectrum (BGS) is obtained, which is used to fit a Lorentzian function to identify the Brillouin frequency of the fiber at each location. Since the Brillouin frequency of the fiber is directly related to the fiber’s strain and temperature, then the strain and/or temperature of the fiber can be easily identified.

One important drawback of the BOTDA-based distributed sensing is its limited spatial resolution of measurements. As will be illustrated in Section 2.2, the BGS obtained from the BOTDA actually reflects the combined SBS effects of the fiber within the spatial resolution of BOTDA and, thus, cannot provide the fiber’s strain at any specific location. It is noted that the spatial resolution of BOTDA is half width of pump pulse light. Moreover, to stimulate the SBS effect, the width of the pump pulse light needs to be larger than the phonon lifetime (i.e., 10 ns), which leads to that the spatial resolution of BOTDA generally cannot be less than 1m. Such a spatial resolution makes it difficult for BOTDA to detect structural cracks, which can only induce large strain in the fiber over very short distance.

**Figure 1:**
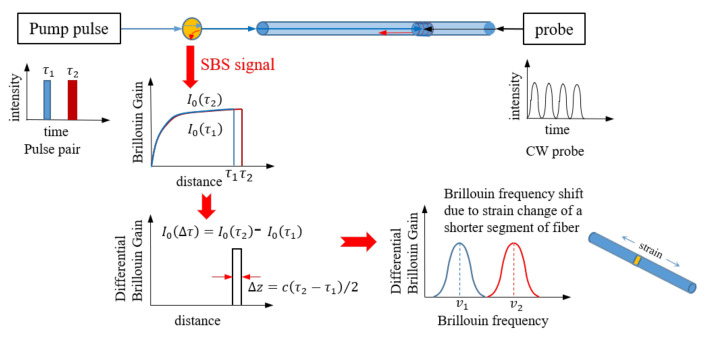
Working mechanism of DPP-BOTDA based distributed strain sensing.

To overcome the drawback of limited spatial resolution, the DPP-BOTDA was proposed. Figure 1 illustrates the working mechanism of the DPP-BOTDA based distributed strain sensing. In DPP-BOTDA system, two separate measurements are implemented using two pump pulse lights with slightly different pulse widths; the subtraction of the Brillouin gain signals from the two pulses are utilized to estimate the fiber’s strain. As shown in Figure 1, the differential Brillouin gain is equivalent to the Brillouin gain of a shorter pulse with the width of $\tau_{2}-\tau_{1}$. Therefore, DPP-BOTDA can effectively reduce the spatial resolution of strain sensing to $c\left(\tau_{2}-\tau_{1}\right)/n/2$, where *c* and *n* are the light speed and the refraction index of fiber. However, it needs pointing out that because the difference of the Brillouin gain signal greatly reduces the signal strength, which decreases the strain sensing accuracy. Therefore, the DPP-BOTDA cannot arbitrarily improve the spatial resolution of strain sensing via making the widths of the two pulses very close to each other. So far, the highest spatial resolution of DPP-BOTDA is reported to be 2 cm by Dong et al. [19]. Compared to the 1-m spatial resolution of BOTDA, DPP-BOTDA greatly improves the spatial resolution of strain sensing, making it possible to detect the occurrence of small structural cracks.

**Figure 2:**
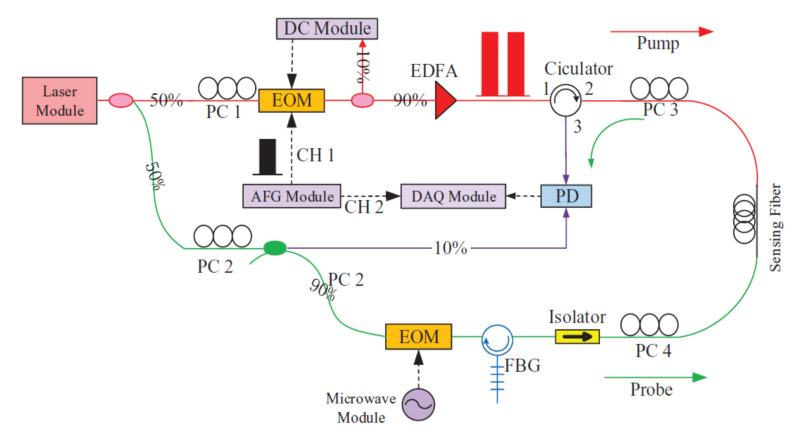
Schematic of the DPP-BOTDA system. PD: photo-detector, PC: polarization controller, EOM: electro-optic modulator, EDFA: Erbium-doped fiber amplifier module, DAQ: data acquisition.

The optical structure of the DPP-BOTDA system used in this paper is shown in Figure 2 [20]. A narrow linewidth 200 kHz fiber laser module with a wavelength of 1550 nm is used as the system light source. The output laser is divided into two channels by a 3dB coupler, which is used as pump light and probe light respectively. A 40 dB high extinction ratio electro-optic modulator (EOM) is used to generate the double pump pulse, which is then amplified by an erbium-doped fiber amplifier (EDFA) before injecting into the sensing fiber. The microwave module modulates the laser through EOM, which is then filtered out the first-order lower sideband by a narrow bandwidth Bragg grating filter. The filtered laser is used as detection light injected into the sensing fiber. Two polarization controllers are used to ensure that the pump light and probe light enter the same axis of the sensing fiber, so the polarization maintaining fiber is more suitable for this system. The Brillouin signal is detected by photoelectric detector and data acquisition module with centimeter level spatial resolution capability.

### 2.2. Theoretical Model of Calculating Brillouin Gain of DPP-BOTDA

As introduced in Section 2.1, both BOTDA and DPP-BOTDA rely on the Brillouin gain of the CW probe light to identify the strain/temperature of the fiber. In this subsection, the theoretical model of calculating the Brillouin gain in the BOTDA, proposed by Bao et al. [21], is briefly reviewed, which can also be used to calculate the Brillouin gain in the DPP-BOTDA system.

Under the steady state condition of the light waves, the Brillouin scattering process can be modeled by using the two coupled light wave equations regarding the pump pulse and the CW probe lights as:

(1) $$\frac{d}{dz}I_{p}\left(z\right)=-g\left(v,v_{B}\right)I_{cw}I_{p}-\alpha I_{p}$$

(2) $$\frac{d}{dz}I_{cw}\left(z\right)=-g\left(v,v_{B}\right)I_{cw}I_{p}+\alpha I_{cw}$$

where $I_{p}\left(z\right)$ and $I_{cw}\left(z\right)$ are the intensities of the pump pulse and CW probe lights at the location z; *z* is the distance from the pulse laser to the optical fiber end; α is the fiber’s attenuation coefficient; $g\left(v,v_{B}\right)$ is the Brillouin gain factor, which can be modelled as a Lorentzian function as:

(3) $$g\left(v,v_{B}\right)=\frac{g_{0}{\left(\Delta v_{B}/2\right)}^{2}}{{\left(v-v_{B}\right)}^{2}+{\left(\Delta v_{B}/2\right)}^{2}}$$

where $v_{B}$ is the Brillouin frequency of the optical fiber; v is the sweeping frequency, defined as the frequency difference between the pump pulse and CW probe lights; $g_{0}$ is the peak Brillouin gain factor; $\Delta v_{B}$ is the Brillouin gain linewidth.

By solving the Equations (1) and (2), the Brillouin gain of the CW probe light at the location of z can be calculated as:

(4) $$\int_{z}^{z+\tau }\frac{dI_{cw}}{I_{cw}\left(z\right)}=\int_{z}^{z+\tau }\left\{-g\left(v,v_{B}\right)I_{p}\left(0\right)\exp\left[-\frac{g\left(v,v_{B}\right)I_{cw}\left(L\right)e^{-\alpha L}\left(e^{\alpha z}-1\right)}{\alpha }-\alpha z\right]+\alpha \right\}dz$$

where τ is the width of the pump pulse light; L is the total length of the optical fiber. If the strain of the fiber keeps constant within the width of the pump pulse light (i.e., from location z to location z+τ), the Brillouin frequency $v_{B}$ of the fiber will remain a constant in the integral region of Equation (4). Then, the integral of Equation (4) can be readily evaluated as:

(5) $$B_{g}\left(z\right)=\frac{I_{cw}\left(z\right)}{I_{cw}\left(z+\tau \right)}=\exp\left\{K_{0}\left[\frac{E_{2}\left(\beta x_{1}\right)}{x_{1}}-\frac{E_{2}\left(\beta x_{2}\right)}{x_{2}}\right]-\alpha \tau \right\}$$

where $B_{g}\left(z\right)$ denotes the Brillouin gain of the fiber at location z due to the SBS’s effects; $x_{1}=e^{\alpha z}$; $x_{2}=e^{\alpha \left(z+\tau \right)}$; $K_{0}=\frac{g\left(v,v_{B}\right)I_{p}\left(0\right)e^{\beta }}{\alpha }$; $\beta =\frac{g\left(v,v_{B}\right)I_{cw}\left(L\right)e^{-\alpha L}}{\alpha }$; $E_{2}\left(\cdot \right)$ denotes the second order exponential integral, defined as:

(6) $$E_{2}\left(x\right)=\int_{1}^{\infty }\frac{exp\left(-xt\right)}{t^{2}}dt$$

The result of Equation (6) provides a simple formula to calculate the Brillouin gain $B_{g}$. Then, by changing different sweeping frequencies, the Brillouin gain at each sweeping frequency v can be calculated and, thus, the whole Brillouin gain spectrum (BGS) is obtained. Via fitting the BGS with a Lorentzian model as show in Equation (7), the Brillouin frequency of the fiber between location z and z+τ can be identified:

(7) $$\underset{{\hat{v}}_{B},\Delta {\hat{v}}_{B},{\hat{g}}_{0}}{\min}J\left({\hat{v}}_{B},\Delta {\hat{v}}_{B},{\hat{g}}_{0}\right)=\overset{n}{\underset{i=1}{\sum }}{\left|\frac{{\hat{g}}_{0}{\left(\Delta {\hat{v}}_{B}/2\right)}^{2}}{{\left(v_{i}-{\hat{v}}_{B}\right)}^{2}+{\left(\Delta {\hat{v}}_{B}/2\right)}^{2}}-{\hat{B}}_{g}\left(v_{i}\right)\right|}^{2}$$

where ${\hat{v}}_{B},\Delta {\hat{v}}_{B},\;{\hat{g}}_{0}$ are the estimated Brillouin frequency, Brillouin gain linewidth and peak Brillouin gain factor, respectively; ${\hat{B}}_{g}\left(v_{i}\right)$ is the measured Brillouin gain by the BOTDA demodulator with the sweeping frequency $v_{i}$. It is noted that although the above calculation method of the Brillouin gain is derived for the BOTDA system, it can be easily extended to the DPP-BOTDA system by changing the lower and upper integration bound of Equation (3) from z and z+τ to $z+\tau_{1}$ and $z+\tau_{2}$, respectively.

### 2.3. Simulation of BGS of Fiber with Non-Uniformly Distributed Strain

In Section 2.2, the method of calculating the Brillouin gain of the fiber with constant strain within the spatial resolution of BOTDA (or DPP-BOTDA) was presented. However, in the real applications of structural health monitoring, the strain in the fiber may not remain constant over the length of the spatial resolution. With non-uniformly distributed strain, the Brillouin gain of the fiber will unavoidably change and cannot be calculated by simply using the result of Equation (5).

In this section, the method of calculating the Brillouin gain of the fiber with non-uniformly distributed strain within the pulse width of the pump pulse light is proposed. To deal with the non-uniform distribution of the strain, the width of the pump pulse light is equally divided into *n* segments. It is assumed that within each segment the fiber’s strain is approximately constant; thus, the Brillouin gain contributed by each segment can be calculated using the result of Equation (5), and the Brillouin gain of the pump pulse light is equal to the summation of these contributions. By adopting this strategy, the Brillouin gain of the fiber with non-uniformly distributed strain could be calculated using Equation (8):

(8) $$B_{g}\left(z\right)=\frac{I_{cw}\left(z\right)}{I_{cw}\left(z+\tau \right)}=\exp\left(-\alpha \tau \right)\exp\left\{\overset{n}{\underset{i=1}{\sum }}\frac{K_{i}}{\alpha }\exp\left(\beta_{i}\right)\left\{\begin{array}{c} \frac{\exp\left(-\beta_{i}x_{i+1}\right)}{x_{i+1}}-\frac{\exp\left(-\beta_{i}x_{i}\right)}{x_{i}}\\ +\beta_{i}\left[E_{1}\left(\beta_{i}x_{i}\right)-E_{1}\left(\beta_{i}x_{i+1}\right)\right] \end{array}\right\}\right\}$$

where:

(9) $$\begin{array}{c} x_{i}=e^{\alpha \left[z+\frac{\tau }{n}\left(i-1\right)\right]},\;K_{i}=g_{i}I_{p}\left(0\right),\;\beta_{i}=\frac{g_{i}I_{CW}\left(L\right)e^{\alpha L}}{\alpha },\;\left(i=1,2,\ldots ,n\right)\\ E_{1}\left(x\right)=\int_{1}^{\infty }\frac{\exp\left(-xt\right)}{t}dt \end{array}$$

where $g_{i}=g_{i}\left(v_{B_{i}},\Delta v_{B}\right)$ is the Brillouin gain factor of the *i*th segment of the fiber; $v_{B_{i}}$ is the Brillouin frequency of the *i*th segment of the fiber, which is related to the strain of the *i*th segment.

**Figure 3:**
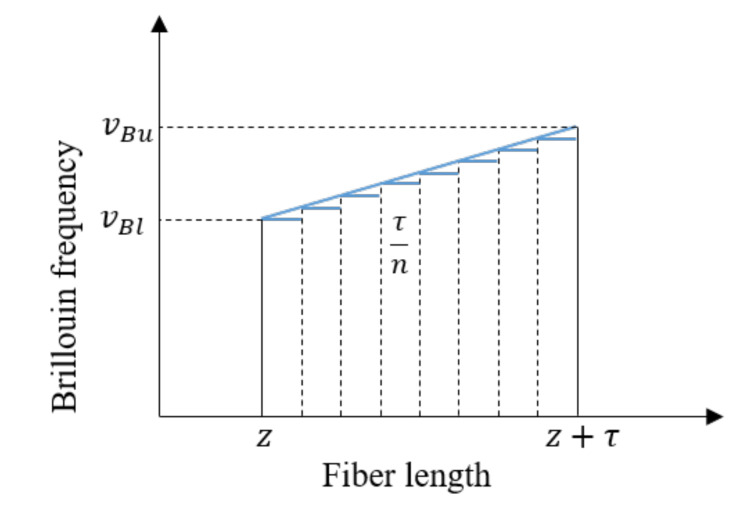
Brillouin frequency of optical fiber with linearly distributed strain field.

**Figure 4:**
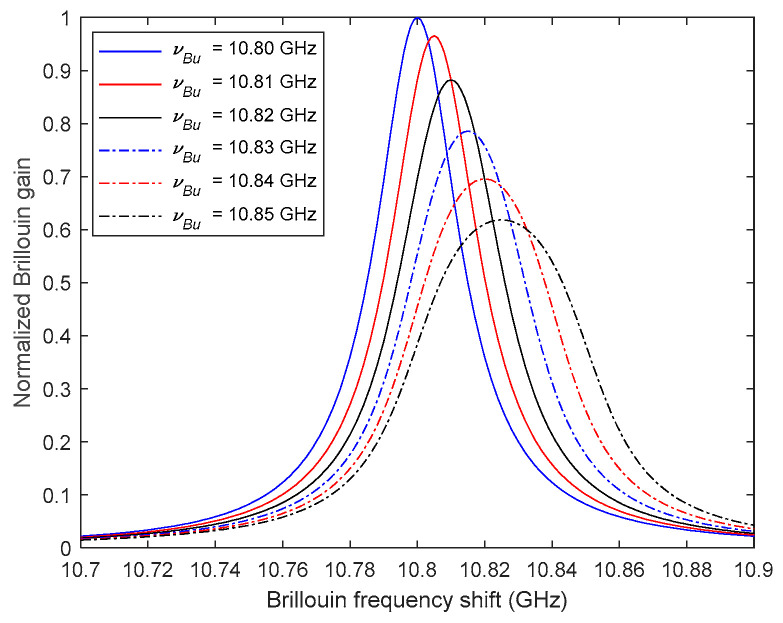
Normalized BGS at location z corresponding to linearly distributed strain fields.

To demonstrate how the non-uniformly distributed strain changes the BGS of the fiber, a numerical example is conducted herein, in which the fiber is subject to linearly changed strain as shown in Figure 3. It is assumed that the fiber’s strain at location z, represented by the value of $v_{Bl}$, remains unchanged and the fiber’s strain at location z+τ, represented by the value of $v_{Bu}$, gradually increases from 10.80 GHz to 10.85 GHz with the increment of 0.01 GHz. Other parameters of the simulation are the same as the numerical example in Section 2.2. The fiber between locations z and z+τ is divided into 100 segments to calculate the Brillouin gain $B_{g}$ using the result in Equation (8). Figure 4 shows the changes of the BGS with different values of $v_{Bu}$, demonstrating that with the increase of the strain gradience in the fiber, the identified equivalent Brillouin frequencies (i.e., ${\hat{v}}_{B}$) increase, along with the decrease of the peak Brillouin gain factor (i.e.,$\;{\hat{g}}_{0}$) and the increase of the Brillouin gain linewidth (i.e., ${\hat{\Delta v}}_{B}$). This result is easily understandable. Because the BGS of the fiber between z and z+τ can be considered as the combination of many small BGSs, each of which is contributed by a small segment of the fiber with its Brillouin frequency. Equations (3) and (4) indicate that if the sweeping frequency v just equals the fiber’s Brillouin frequency $v_{B}$, the SBS effect is maximized. Therefore, when the fiber strain within the width of the pump pulse light is uniformly distributed, the peaks of all small BGSs locate at the same Brillouin frequency, leading to that the combined BGS has the highest Brillouin peak gain factor and the narrowest Brillouin gain linewidth. On the contrary, when strain is not uniformly distributed, the peaks of the small BGSs spread, which reduces the peak Brillouin gain factor and also increases the Brillouin gain linewidth of the combined BGS.

## 3. Detect Structural Cracks via DPP-BOTDA

In this section, the structural crack induced fiber strain is investigated, the results of which are used to simulate the BGS in the fiber obtained by the DPP-BOTDA when a structural crack is present. Based on the simulation results, the irregular features of the BGS due to structural cracks are summarized, which will be used to investigate the structural crack detection and quantification method in Section 3.4 and Section 3.5

### 3.1. Crack-Induced Strain in Optical Fiber

In order to check the ability of the DPP-BOTDA system to detect the occurrence of structural cracks, the BGS obtained by these systems need to be simulated, which requires the strain distribution in the fiber due to structural crack. Many researches have investigated the mechanism of strain transfer from the structure surface to the sensing fiber when a structural crack is present. Ansari and Libo [22] proposed a shear transfer model to analyze the strain distribution of the sensing fiber. Feng et al. [11] studied the shear transfer models, considering the protective coating of the fiber being in both elastic and elasto-plastic stages. Wan et al. [23] developed a shear transfer model of the bare fiber directly sticked to the structural surface.

**Figure 5:**
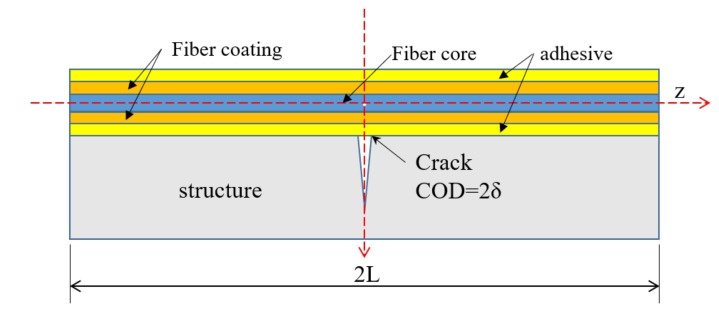
Illustration of strain field of fiber with a structural crack.

In this article, the elastic shear transfer model of the fiber proposed by Feng [11] is adopted, the configuration of which is illustrated in Figure 5. The optical fiber with outer coating is sticked to the structural surface with the adhesive. The bonding length between the fiber and the structure is 2L. It is assumed that the structure has a constant strain $\varepsilon_{m}$ and also has a surface crack with crack open distance (COD) of 2 δ at the location *z* = 0. The strain distribution $\varepsilon_{f}\left(z\right)$ of the fiber core in linear elastic stage can be given by Equation (10):

(10) $$\varepsilon_{f}\left(z\right)=C_{1}\gamma \exp\left(\gamma z\right)-C_{2}\gamma \exp\left(-\gamma z\right)+\varepsilon_{m}$$

where γ is the shear lag factor of the fiber, which is related to the geometric and material properties of the fiber coating and the adhesive layer; $C_{1}$ and $C_{2}$ are constants that can be determined from the boundary conditions, which are given as for z>0:

(11) $$C_{1}=-\frac{\delta }{\exp\left(2\gamma L\right)+1}$$

(12) $$C_{2}=-\frac{\delta \exp\left(2\gamma L\right)}{\exp\left(2\gamma L\right)+1}$$

The COD is generally very small (i.e., on the order of $10^{-4}$ m), which is much smaller than $\exp\left(2\gamma L\right)$; thus, $C_{1}\approx 0$ and $C_{2}\approx -\delta$. Then, the strain in the fiber for z>0 can be simplified as:

(13) $$\varepsilon_{f}\left(z\right)\approx \delta \gamma \exp\left(-\gamma z\right)+\varepsilon_{m}$$

Moreover, due to the symmetry with respect to the crack configuration, the strain distribution of the fiber core can be written using the following stepwise function:

(14) $$\varepsilon_{f}\left(z\right)\approx \left\{\begin{array}{cc} \delta \gamma \exp\left(-\gamma z\right)+\varepsilon_{m} & z>0\\ \delta \gamma \exp\left(\gamma z\right)+\varepsilon_{m} & z\leq 0 \end{array}\right.$$

**Figure 6:**
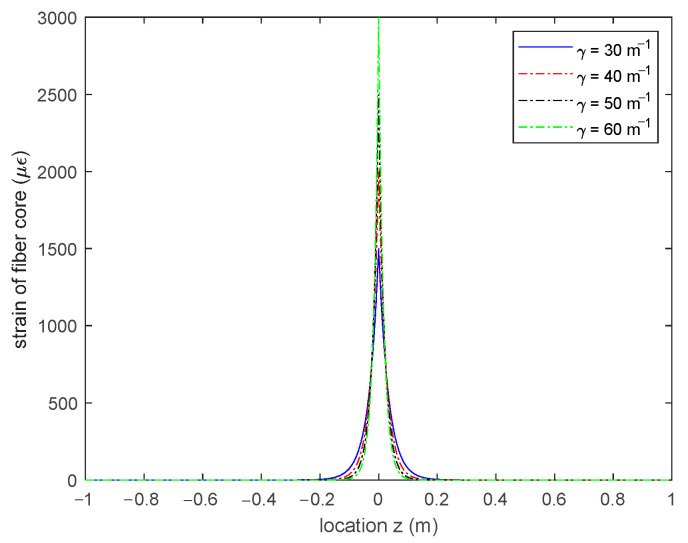
Strain distribution of fiber core with different shear lag factors (COD = 0.1 mm and $\varepsilon_{m}=0\;\mu \varepsilon$).

Equation (14) demonstrates that the crack-induced fiber strain can be simply determined by two parameters: COD 2δ and the shear lag factor γ. Figure 6 shows the strain distribution of the fiber core with different shear lag factors under the condition that 2δ=0.1 mm and $\varepsilon_{m}=0\;\mu \varepsilon$, from which it can be clearly seen that the larger the shear lag factor, the sharper the strain distribution will be at the location of the crack.

### 3.2. Simulation of Crack-Induced BGS Measured by DPP-BOTDA

Combining the crack-induced fiber strain distribution obtained in Section 3.1 with the BGS calculating method with non-uniformly strain in the fiber proposed in Section 2.3, the crack-induced BGS of the fiber can be simulated. In this subsection, a numerical example is conducted to analyze the characteristics of crack-induced BGS of the fiber obtained by the DPP-BOTDA system.

**Figure 7:**
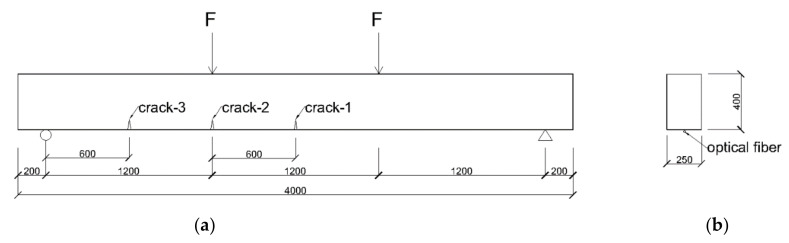
Setup of numerical example (**a**) side view of RC beam (**b**) cross section of RC beam.

**Figure 8:**
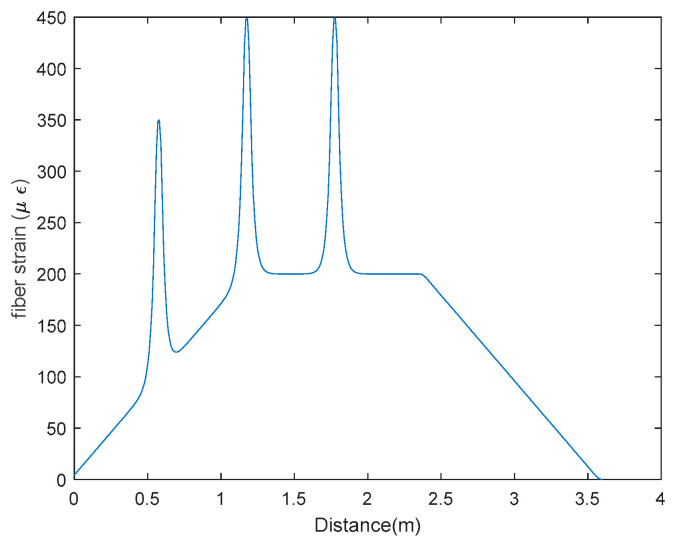
Strain distribution of fiber core with structural cracks (COD = 0.02 mm and $\gamma =40\;m^{-1}$).

In the example, the structure is a simple-supported RC beam with the span of 3.6 m, which is shown in the Figure 7. Two vertical point loads of the same magnitude are applied on the top of the beam at the location of 1200 mm and 2400 mm, respectively, from the left support. The cross section of the beam is 250 × 400 mm. Three cracks are considered to occur on the bottom of the RC beam, which occur at the locations of 600 mm, 1200 mm and 1800 mm from the left support. The reason of selecting these crack locations is that prior to the occurrence of structural cracks, the structural strain near the location of crack-1 is constant and while, the structural strains near crack-3 are linearly changed and the structural strains near crack-2 have a transition, which provide more realistic scenarios of structural strain distribution to test the feasibility of the proposed crack detection method. The strain of the fiber, after the occurrence of the cracks, are simulated by adding the crack-induced additional strain, illustrated by Equation (14), to the original structural strain. Figure 8 shows the strain distribution in the fiber after the occurrence of cracks. It is assumed in the simulation that the cracks occur when the largest tensile strain on the beam bottom reaches the ultimate tensile strain of the concrete (i.e., about 200 με) and the crack width (i.e., COD) is 0.02 mm with the shear lag factor γ of 40 m^−1^.

**Figure 9:**
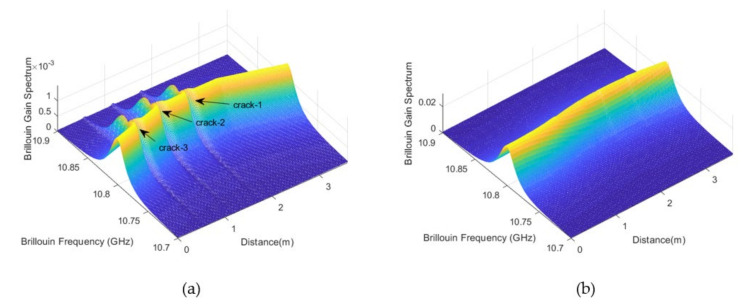
Comparison of Brillouin gain spectrum obtained by DPP-BOTDA and BOTDA systems (**a**) DPP-BOTDA (**b**) BOTDA.

To simulate the BGS obtained by the DPP-BOTDA system, the following parameters are selected for the DPP-BOTDA: L=100 m, z=50 m, $v_{B}=10.80\;\mathrm{GHz}$, $I_{p}\left(0\right)=30\;\mathrm{mW}$, $I_{CW}\left(100\right)\;=5\;\mathrm{mW}$, $\alpha =4.15\times 10^{-5}\;m^{-1}$; the widths of the two pump pulse lights are $\tau_{1}=2.0\;m$ and $\tau_{2}=2.1\;m$, respectively, which makes the spatial resolution of the DPP-BOTDA be 5 cm. The DPP-BOTDA system provides a strain measurement every one centimeter along the fiber. Figure 9a shows the BGS of the fiber obtained by the DPP-BOTDA system, from which it can be seen that the three cracks induced large local abnormal strain distribution is clearly detected. Moreover, as the target for comparison, the BGS from the BOTDA system is also simulated. The width of pump pulse light is 2 m, which makes the spatial resolution of the BOTDA system be 1m. As shown in Figure 9b, due to the limited spatial resolution, the structural cracks cannot be detected by the BOTDA system. However, it needs pointing out that the magnitude of the BGS obtained by the DPP-BOTDA is much smaller than that of the BOTDA, which makes the BGS results from the DPP-BOTDA more susceptible to noise disturbance.

### 3.3. Characteristics of Crack-induced BGS

Moreover, to investigate how the BGS, obtained by the DPP-BOTDA, changes with different width of structural cracks, a lot of simulations have been conducted using the RC beam described in Section 3.3. To simulate the development of the structural crack, the forces applied on the beam gradually increases. In this study, it is simply assumed that when the forces induced structural strain reaches 200, 400, 600, 800 and 1000 με, the widths of the three cracks increase from 0.02 mm to 0.10 mm with the increment of 0.02 mm.

**Figure 10:**
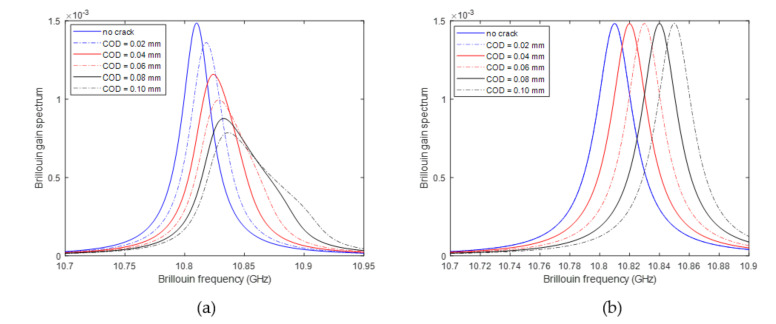
Changes of BGS of fiber at location of (**a**) with crack (**b**) without crack.

Figure 10 shows the BGSs of the fiber at the locations of 1.8 m, where the 1st crack develops, and at the location of 2.2 m, where no crack is present, demonstrating that the BGS changes of the fiber at the location with and without structural crack are significantly different. When no structural crack is present, the bell-shape BGS simply moves to one direction without shape change; while there is a crack, the BGS shape changes drastically: with the increased width of structural crack, the peak of the BGS becomes much smaller and, meanwhile, the BGS becomes much wider.

To quantitatively reflect the above features of the BGS changes, the parameters of Brillouin frequency ${\hat{v}}_{B},$ peak Brillouin gain factor ${\hat{g}}_{0}$, Brillouin gain linewidth $\Delta {\hat{v}}_{B}$ need to be determined. However, it is noted in Figure 10a that due to drastic change of fiber strain near the crack, the BGS at the crack location shows an asymmetric change and is no longer a symmetric Lorentzian function. Thus, using a Lorentzian model to fit the BGS at the crack will lead to large error of the estimated parameters. Therefore, the following procedure is used to estimate the above parameters of the BGS. The peak Brillouin gain factor ${\hat{g}}_{0}$ and Brillouin frequency ${\hat{v}}_{B}$ are determined by the maximum value of the BGS as well as the corresponding sweeping frequency; the Brillouin gain linewidth $\Delta {\hat{v}}_{B}$ is determined by the difference of the two sweeping frequencies, the magnitudes of which are equal to the half of peak Brillouin gain factor ${\hat{g}}_{0}$. It is noted that if the BGS can be modelled a symmetric Lorentzian function, the above proposed estimation procedure will also provide the similar results as fitting the BGS with a Lorentzian model; but if the BGS is asymmetric, the proposed procedure will give more accurate results.

**Figure 11:**
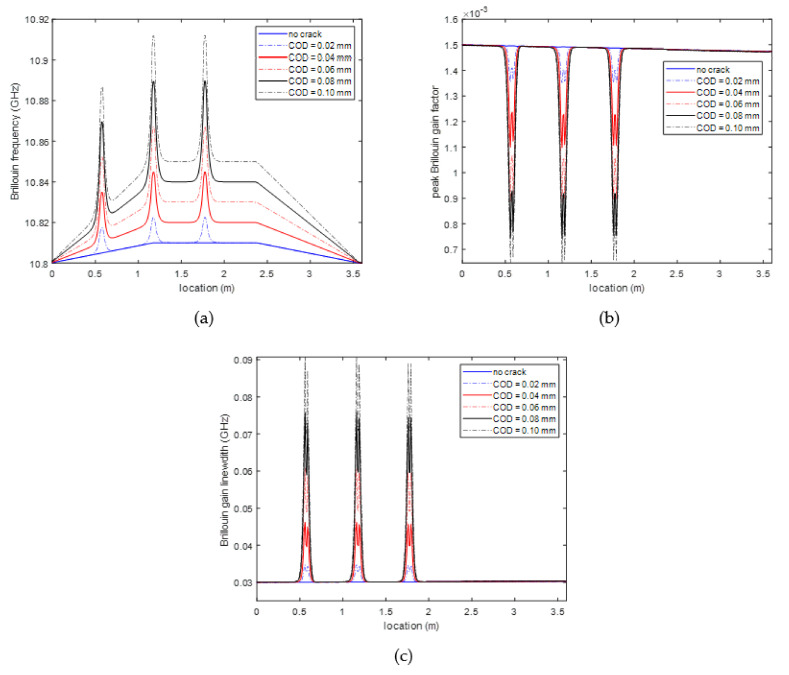
Identified Lorentzian model parameters along the fiber (**a**) Brillouin frequency (**b**) peak Brillouin gain factor (**c**) Brillouin gain linewidth.

The estimated parameters of Brillouin frequency ${\hat{v}}_{B},$ peak Brillouin gain factor ${\hat{g}}_{0}$, Brillouin gain linewidth $\Delta {\hat{v}}_{B}$ along the fiber are plotted in Figure 11. 

Comparing the estimated parameters at the locations with and without structural crack, it can be clearly observed that (1) on one hand, no matter whether the structural crack presents, the estimated Brillouin frequencies change, due to the change of structural strain; (2) on the other hand, both peak Brillouin gain factor and Brillouin gain linewidth are almost unchanged when no structural crack presents, and drastically decreases and increases respectively when the fiber is close to the crack. More importantly, the changes of these two parameters are mainly determined by the presence of structural crack and not related to the values of the structural strain, which makes it possible to utilize these two parameters to construct a structural strain independent crack detect indicator.

### 3.4. Structural Crack Indicator

Based on the above analysis result about the characteristics of crack induced BGS, two parameters, $I_{g_{0}}$ and $I_{\Delta v_{B}}$, are proposed in Equation (15) to quantify the changes of the peak Brillouin gain factor $g_{0}\;$and Brillouin gain linewidth $\Delta v_{B}$, respectively, due to the occurrence of structural cracks:

(15) $$I_{g_{0}}=\frac{\left|g_{0}^{\left(u\right)}-g_{0}^{\left(d\right)}\right|}{g_{0}^{\left(u\right)}},\;I_{\Delta v_{B}}=\frac{\left|\Delta v_{B}^{\left(u\right)}-\Delta v_{B}^{\left(d\right)}\right|}{\Delta v_{B}^{\left(u\right)}}$$

where $g_{0}^{\left(u\right)}$ and $g_{0}^{\left(d\right)}$ are the estimated peak Brillouin gain factor obtained before and after structural cracks develop; $\Delta v_{B}^{\left(u\right)}$ and $\Delta v_{B}^{\left(d\right)}$ are the estimated Brillouin gain linewidth obtained before and after structural cracks develop.

**Figure 12:**
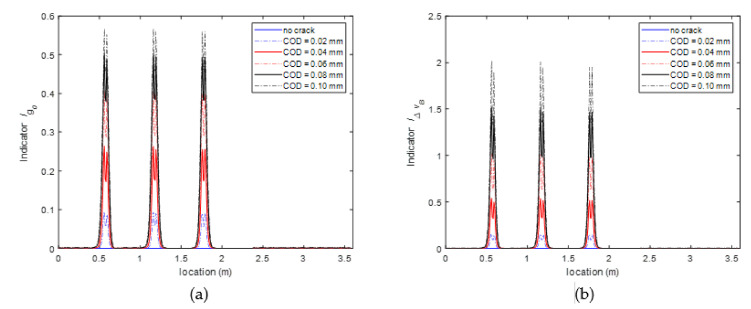
Parameter values of $I_{g_{0}}$ and $I_{\Delta v_{B}}$ along the fiber with different structural crack widths (**a**) $I_{g_{0}}$ (**b**) $I_{\Delta v_{B}}$.

Figure 12a,b show how parameters $I_{g_{0}}$ and $I_{\Delta v_{B}}$ change along the fiber with different widths of structural cracks. It can be seen that both parameters remain close to zero everywhere except at the locations adjacent to the structural cracks, where the parameters increase with the increment of structural crack width. More importantly, the changes of these indicators are only related to the structural crack width, and are independent of structural strain, which make them suitable to infer the occurrence of structural cracks.

Based on the properties of the parameters $I_{g_{0}}$ and $I_{\Delta v_{B}}$, a structural crack indicator is proposed in Equation (16):

(16) $$I=\frac{I_{g_{0}}}{I_{g_{0}}+I_{\Delta v_{B}}}I_{g_{0}}+\frac{I_{\Delta v_{B}}}{I_{g_{0}}+I_{\Delta v_{B}}}I_{\Delta v_{B}}$$

**Figure 13:**
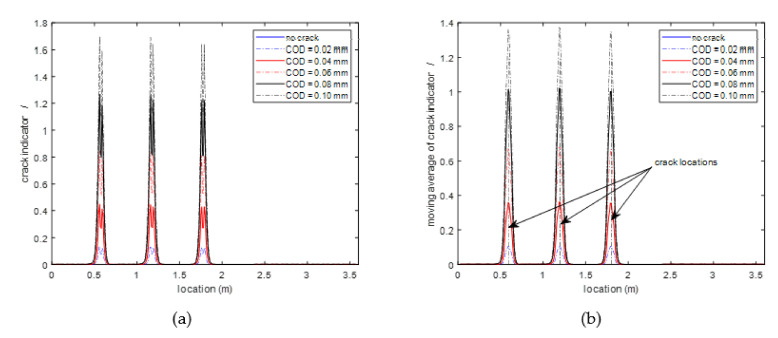
Crack indicator and moving averaged crack indicator along the fiber with different widths of structural cracks (**a**) crack indicator (**b**) moving average of crack indicator.

Figure 13a shows how the proposed crack indicator changes along the fiber with different widths of structural cracks. As predicted, the crack indicator is close to one everywhere except for the regions adjacent to structural cracks, which makes it a good indicator to detect structural cracks. However, as shown in Figure 13a the crack indicators have two peaks near each crack location and the locations of the two peaks are approximately symmetric with the crack location. This phenomenon may be due to the facts that the strains in the fiber change drastically near the structural crack and are approximately symmetric with respect to the crack location. This two-peak phenomenon makes it difficult to use the crack indicator to directly pinpoint the crack location. To cope with this issue, a 5-point moving average window is used to smoothen the crack indicators, the result of which is shown in Figure 13b. The 5-point moving average operation is aiming at smoothening the drastic change of the crack indicators to obtain an averaged value of the crack indicators, which generally should be more robust and stable than the crack indicator at any single point. Moreover, due to the approximate symmetric distribution of the two peaks with respect to the crack location, the 5-point moving averaged crack indicators should be approximately symmetric with a peak value near the crack location. The results of the moving averaged crack indicators in Figure 13b just comply with the above expectation.

### 3.5. Prediction of Structural Crack Width Based on Characteristics of BGS

The width of structural crack is of great importance for evaluating structural safety and durability. However, due to the limitation of the spatial resolution, the strain measurements from the DPP-BOTDA are not directly related to structural crack width. In this section, a method is proposed to predict the structural crack width, based on the characteristic parameters of BGS investigated in Section 3.4.

**Figure 14:**
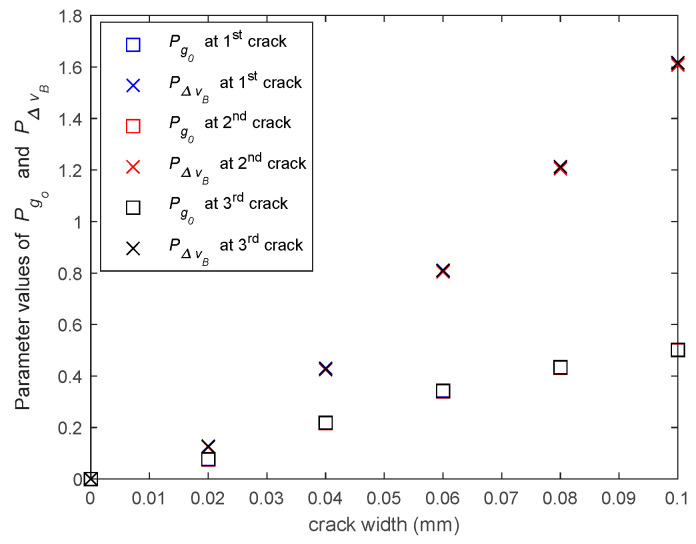
Values of $P_{g_{0}}$ and $P_{\Delta v_{B}}$ at different crack locations with different crack widths.

As shown in Section 3.4, the parameters $I_{g_{0}}$ and $I_{\Delta v_{B}}$ are related to not only the locations but also the widths of structural cracks. Hence, it is possible to use these parameters to formulate a model to predict the structural crack width. Similar to the smoothening operation of crack indicator in Section 3.4, the 5-point moving averages of $I_{g_{0}}$ and $I_{\Delta v_{B}}$ (i.e., $P_{g_{0}}$ and $P_{\Delta v_{B}}$) are first computed, which provide a single peak for each crack. Figure 14 shows how the values of $P_{g_{0}}$ and $P_{\Delta v_{B}}$ at the crack locations changes with different width of structural cracks, demonstrating that these peak values are closely related to the structural crack width. Moreover, Figure 14 also shows that for different structural cracks, the parameters $P_{g_{0}}$ and $P_{\Delta v_{B}}\;$are also very closed at the same width, indicating that the prediction models of structural crack width are almost independent of structural stain, and it is possible to construct a uniform crack width prediction model for all cracks.

In this paper, a linear and a quadratic polynomial model, shown in Equations (17a) and (17b) respectively, are considered to model the relation between the crack width and the parameters $P_{g_{0}}$ and $P_{\Delta v_{B}}$.

(17a) $$\Delta =a_{1}P_{g_{0}}+a_{2}P_{\Delta v_{B}}+a_{3}+\varepsilon$$

(17b) $$\Delta =a_{1}P_{g_{0}}+a_{2}P_{\Delta v_{B}}+a_{3}P_{g_{0}}^{2}+a_{4}P_{\Delta v_{B}}^{2}+a_{5}P_{g_{0}}P_{\Delta v_{B}}+a_{6}+\varepsilon$$

where $P_{g_{0}}$ and $P_{\Delta v_{B}}$ are the peak values of the moving averaged $I_{g_{0}}$ and $I_{\Delta v_{B}}$, respectively, at the crack locations, which are calculated from the measured BGS; Δ denotes the structural crack width; $\left[\begin{array}{ccc} a_{1} & \cdots & a_{n} \end{array}\right]$ denotes the coefficient parameters of the model; ε denotes the model prediction error, which is assumed to be a zero-mean Gaussian random viable with unknown variance $\sigma^{2}$. The maximum likelihood estimation method is adopted to estimate the model parameters $\left[\begin{array}{ccc} a_{1} & \cdots & a_{n} \end{array}\right]$ and the variance of the prediction error ε.

To determine which model should be used, the finite sample corrected Akaike’s Information Criterion (AICc) [24] was adopted, which accounts for both the goodness of data fit and the model complexity with finite sample data. The model giving the smaller AICc value will be selected. Using the data of all three cracks in the numerical example, the variance $\sigma^{2}\;$of the prediction error and the AICc value of the two models were computed and listed in Table 1. Clearly, the quadratic model in Equation (17b) has a slightly smaller AICc value. Thus, it was selected as the crack width prediction model in this numerical example.

**Figure 15:**
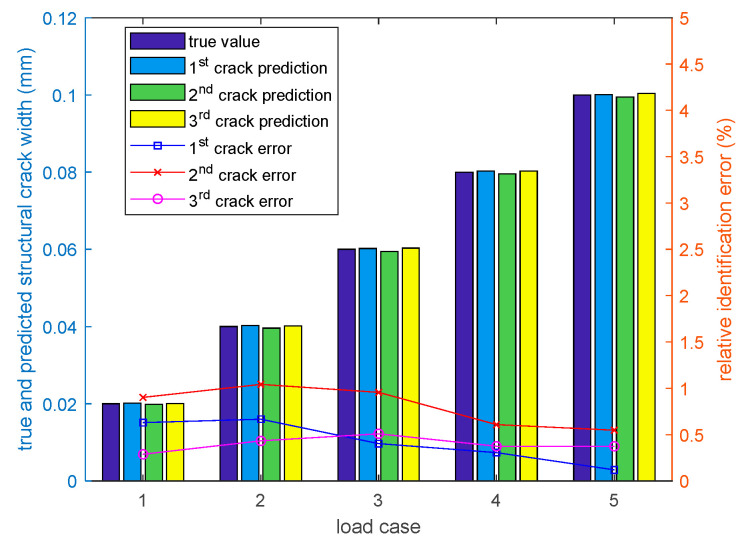
Comparison of the true and predicted crack widths.

Figure 15 shows the comparison of the true and predicted crack widths, illustrating that the prediction model, the developed crack prediction model can very accurately predict the widths of all cracks, regardless of the original structural strain at the crack location.

### 3.6. Discussion

**Figure 16:**
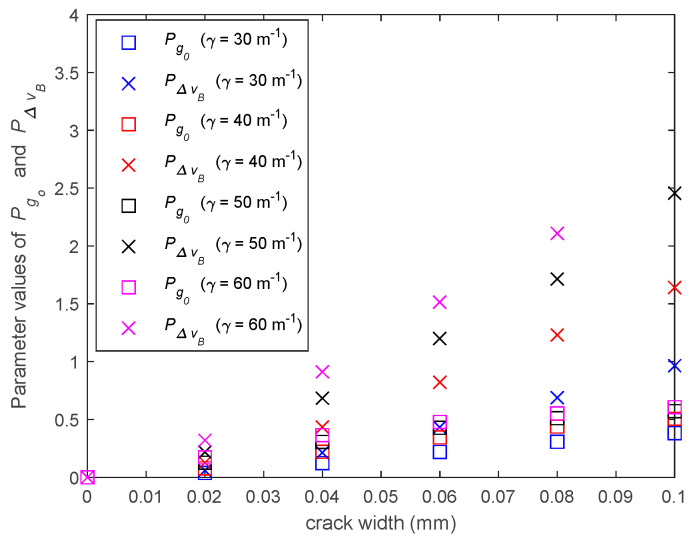
Values of $P_{g_{0}}$ and $P_{\Delta v_{B}}$ at crack-1 with different shear lag factors.

To explain the reason that the proposed crack width prediction model is independent of structural stain, the characteristics of parameters $I_{g_{0}}$ and $I_{\Delta v_{B}}$ are first reviewed. As shown in Section 3.4, $I_{g_{0}}$ and $I_{\Delta v_{B}}$ reflect how the peak Brillouin gain factor decreases and the Brillouin gain linewidth widens after structural cracks occur. The peak values of moving average of $I_{g_{0}}$ and $I_{\Delta v_{B}}$ at the crack location are only determined by the smoothness of the strain distribution in the fiber near the crack, which is dominated by the crack-induced strain. Therefore, if the model of crack-induced strain in the fiber is the same along the fiber, which is equivalent to the same shear lag factor γ along the fiber, the model in Equation (17a) or (17b) can be used to predict the width of all cracks along the fiber based on the BGS measurements. But if the model of crack-induced strain changes, the crack width prediction model will also change. To illustrate this result, the BGSs of the fiber are re-simulated with different shear lag factors; and the input parameters $P_{g_{0}}$ and $P_{\Delta v_{B}}$ of the crack width prediction model of the 1st crack are computed from the simulated BGS and plotted in Figure 16. It can be seen that when the shear lag factors are different, parameters $P_{g_{0}}$ and $P_{\Delta v_{B}}$ will be different even for the same crack with the same width. Consequently, the crack width prediction model will be different.

## 4. Experimental Investigation

In order to verify the proposed methods to detect structural crack as well as estimate the structural crack width, an experiment was conducted on a simple supported RC beam in this study.

### 4.1. Experimental Setup

**Figure 17:**
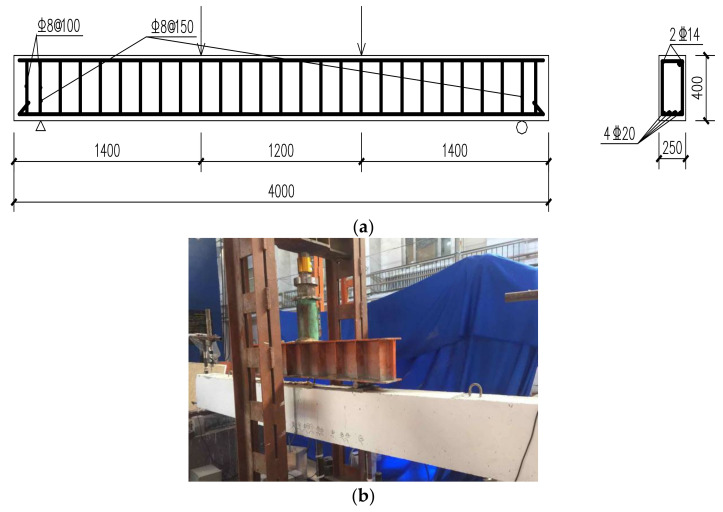
Setup of experiment (**a**) configuration of the tested RC beam (**b**) picture of the tested beam.

**Figure 18:**
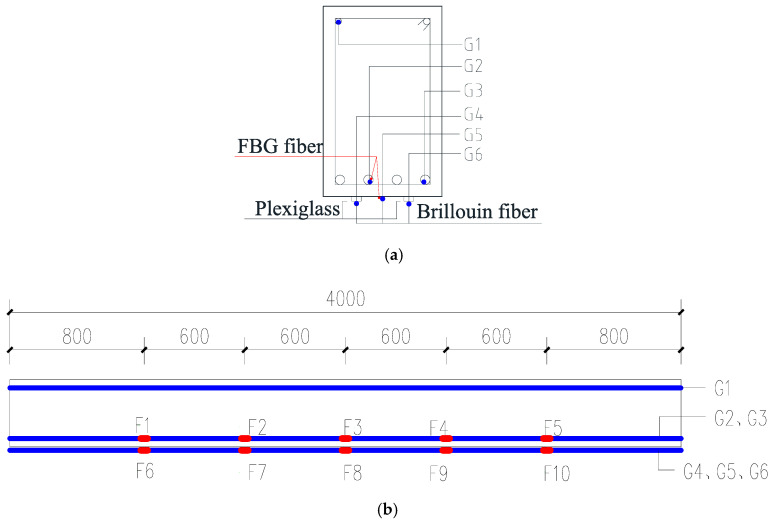
Layout of Brillouin and FBG optical fiber sensors (**a**) Brillouin fiber sensor (**b**) FBG sensors.

The experimental setup is shown in Figure 17. The total length of the beam is 4 m, and the two supports are 0.2 m away from the beam ends. The beam is subject to 2-point loads of the same magnitude. To monitor the structural strain of the tested beam during the experiment, totally six optical fibers are installed inside and on the bottom surface of the beam, as shown in Figure 18. Three fibers (i.e., G1, G2 and G3) are attached to longitudinal steel bars by epoxy; and the others (i.e., G4, G5 and G6) are sticked to the bottom surface of the RC beam. For the fibers installed with steel bars, the strain of the steel bars along the whole length of the beam are measured; for the fibers sticked on the bottom of the beam, due to the interference of the beam supports, the fibers cannot be installed along the whole length of the beam and, thus, only the stains of the 2.4 m-long middle segment of the beam are measured. Moreover, two structural surface sticking methods for optical fibers are adopted herein to investigate their effects on structural strain transition: G5 fiber is directly sticked to concrete surface by epoxy, and G4 and G6 fibers are first sticked to a 2 mm thick Plexiglass sheet, which is sticked to the concrete surface. The six fibers are serially connected, which is then connected to the DPP-BOTDA demodulator. To verify the accuracy of the measured strains by the DPP-BOTDA, two addition fibers, each of which has five fiber Bragg grating (FBG) strain sensors, are installed along with the fibers G2 and G5; the locations of the FBG sensors are labeled with F1 to F10 and shown in Figure 18b.

**Figure 19:**
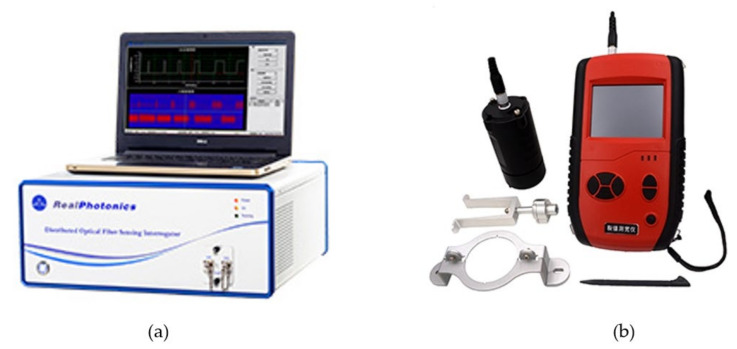
Measuring equipment (**a**) RP1002 high spatial resolution DPP-BOTA demodulator (**b**) ZP-CK103 structural crack width meter.

**Figure 20:**
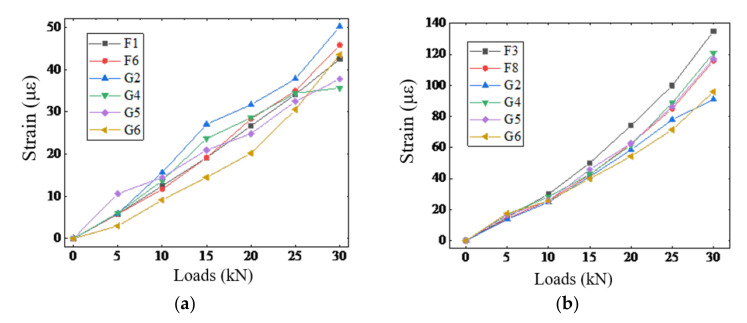
Comparison of strain measurements from DPP-BOTDA and FBG (**a**) at locations of F1 and F6 (**b**) at locations of F3 and F8.

In the experiment, the RP1002 high spatial resolution DPP-BOTDA demodulator, manufactured by Realphotonics^©^ (Anshan, China), is utilized to measure the distributed structural strain in the beam. This demodulator, shown in Figure 19a, can reach the spatial resolution of 2 cm for distributed strain and temperature sensing, which is the highest among similar DPP-BOTDA equipment around the world. However, to obtain higher SNR signals, the spatial resolution is set to be 5 cm in the experiment. The RP1002 demodulator gives a strain reading every 1 cm. To measure the strain in the FBG sensor, the Tw-si255 fiber grating demodulator was used, the resolution of which reaches 1pm. Furthermore, the ZP-CK103 structural crack meter, shown in Figure 20b, is used to measure the width of concrete surface crack during the experiment, which provides the crack measurement accuracy around 0.01 mm.

### 4.2. Experimental Results

During the experiment, the loads applied on the beam gradually increased with 20 kN increment each level. Under each load level, the strains of the tested beam were measured three times by the RP1002 DPP-BOTDA and Tw-si255 fiber grating demodulators; and the average of the measurements was used for the data analysis.

Figure 20 compares of the strain measurements obtained from DPP-BOTDA and FBG demodulators before the occurrence of structural cracks, demonstrating that the strains measured by DPP-BOTDA match relatively well with those obtained by the FBG sensors, particularly at the locations of F3 and F8 where the structural strain is uniform.

**Figure 21:**
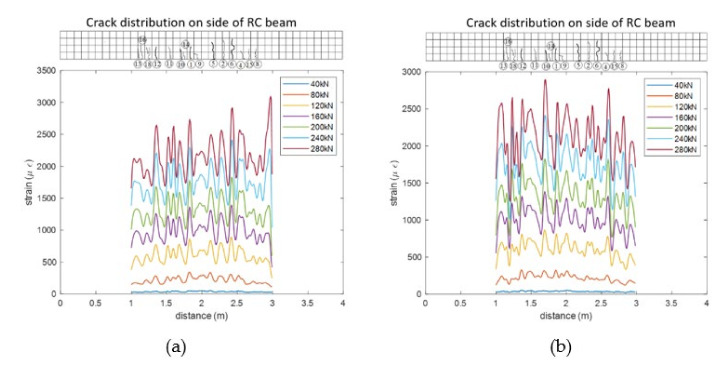
Strain distribution in the fibers under different load levels (**a**) fiber G4 (**b**) fiber G5.

Figure 21 shows the strain distribution along the fibers G4 and G5 that are sticked to the bottom surface of the beam using different sticking methods. It can be seen that when the load level reaches 80 kN, the original flat strain distribution in the middle part fiber begin to fluctuates, which indicates the occurrence of the structural cracks. 

**Figure 22:**
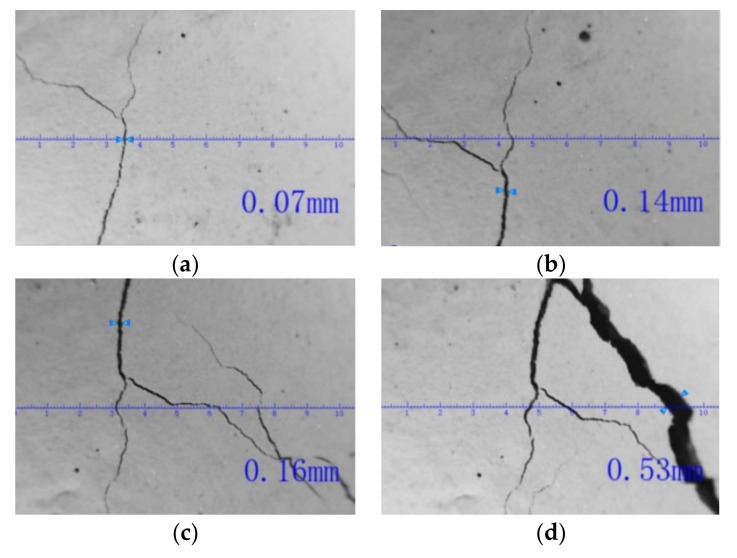
Development of No. 5 crack under different loads (**a**) 80 kN (**b**) 200 kN (**c**) 240 kN (**d**) 280 kN.

During the loading test, the initiation and development of the cracks on the concrete surface were carefully checked and recorded. It was found that most structural cracks begin to form at the load level of 80 kN, which corresponds to the results of the strain measurements. During the process of the test, totally 18 structural cracks were found on the side of the beam, and the width of every crack under different load levels were recorded and listed in Table 2. Figure 22 shows the pictures of No. 5 structural cracks under the load levels of 80, 200, 240 and 280 kN.

### 4.3. Verification of Proposed Structural Crack Identification Method

**Figure 23:**
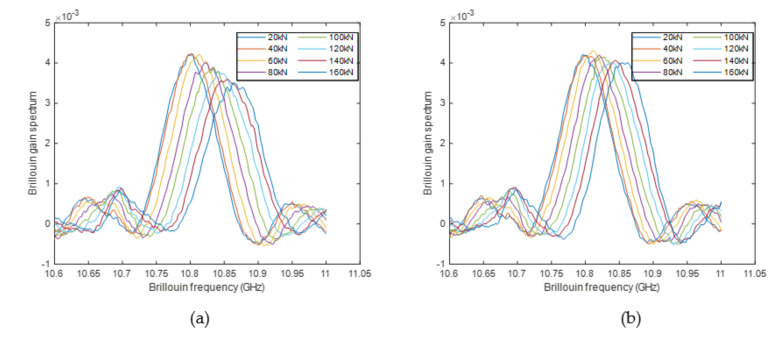
Comparison of BGS under different load levels (**a**) with crack (**b**) without crack.

To verify the structural crack detection method proposed in Section 3.4, the BGSs at the locations with and without structural crack under different load levels are plotted in Figure 23. The similar phenomenon, obtained in the simulation, was also observed. When no structural crack is present, the shape of the BGSs almost does not change and simply moves to one direction; while a crack occurs, the BGS shape changes drastically: with the increase of crack width, the peak of the BGS significantly reduces and the linewidth of the BGS becomes much larger. Therefore, it is expected that the cracked indicator in Equation (16), which was proposed based on the above features of the crack-induced BGS changes, can well reflect the occurrence of true structural cracks.

**Figure 24:**
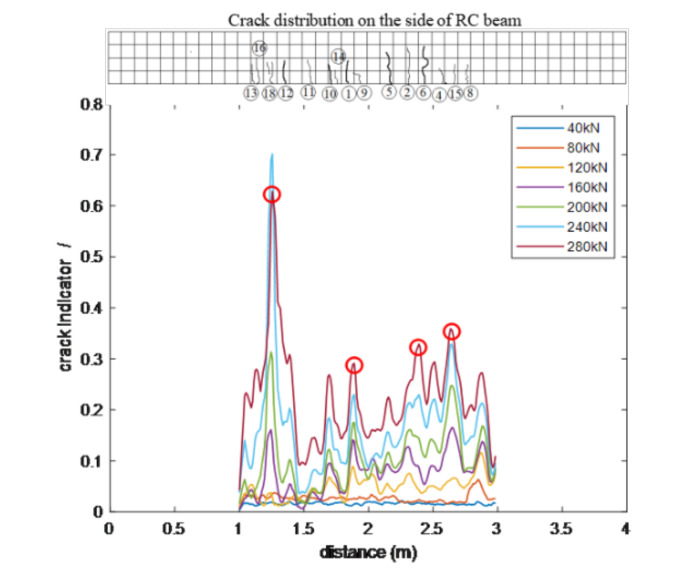
Crack indicator along fiber G5 under different load levels.

Based on the measured BGSs under different load levels, the crack indicator in Equation (16) along the fibers under different load levels was calculated. Figure 24 shows the changes of the crack indictor along the fiber G5, illustrating that the proposed crack indicator clearly detects the occurrence of structural crack as early as the load level just reaches 120 kN.

**Figure 25:**
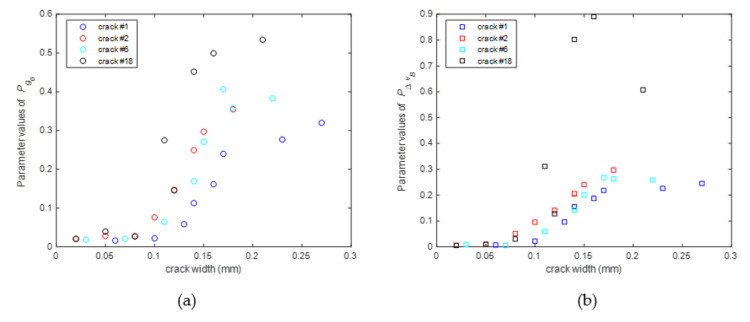
Relations between parameters $P_{g_{0}}$ & $P_{\Delta v_{B}}$ and crack widths of four major cracks (**a**) $P_{g_{0}}$ (**b**) $P_{\Delta v_{B}}$.

In Section 3.5, a structural crack width prediction model was proposed based on the features of crack-induced BGS changes. In this experiment, the measurement results of four major cracks (i.e., crack #1, #2, #6 and #18) are used to verify the effectiveness of proposed crack width prediction method. The location of the four cracks are marked with red circles in Figure 24. Using the measured BGS, the parameters $P_{g_{0}}$ and $P_{\Delta v_{B}}$, used to quantify the crack-induced changes of the peak Brillouin gain factor and the Brillouin gain linewidth, were calculated. Figure 25 shows the relations between the parameters $P_{g_{0}}$ and $P_{\Delta v_{B}}$ and the widths of the four cracks. The positive correlation between the parameters $P_{g_{0}}$ and $P_{\Delta v_{B}}$ and the crack width are clearly observed, which, however, are not as simple as those shown in the numerical examples. There are possible two reasons for this result. First, due to the safety concern, the persons who carried out the experiment did not go under the beam to measure the crack width directly at the fiber location. Instead, they measured the cracks on the side of beam near the beam bottom. Although the cracks on the side of the beam should be closely related to those at bottom, their width are definitely different. It likely contributes to the above result. Second, there are several minor cracks near these major cracks, which will change the strain distribution in the fiber near crack location. It unavoidably changes the measured BGS as well as the consequently derived parameters $P_{g_{0}}$ and $P_{\Delta v_{B}}$.

**Figure 26:**
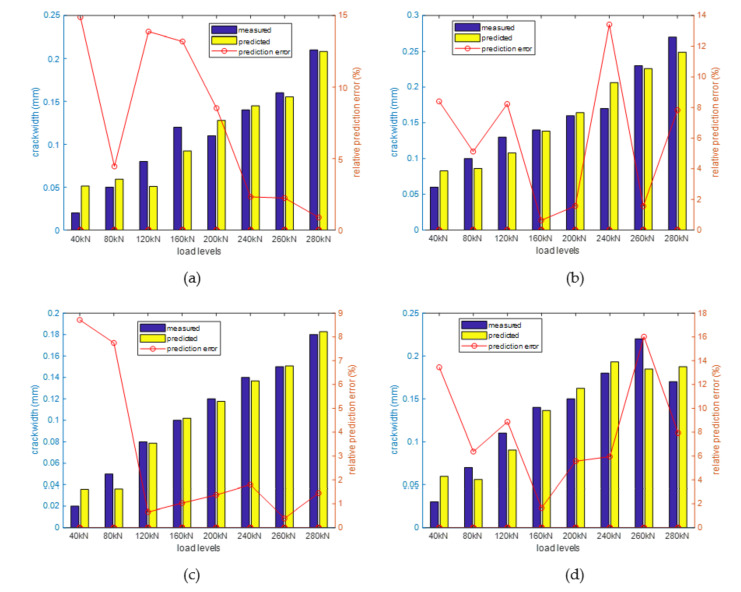
Comparison of the measured and predicted crack widths under different load levels in the experiment (**a**) crack No. 18 (**b**) crack No. 1 (**c**) crack No. 2 (**d**) crack No. 6.

Due to the above analyzed reason, the crack width prediction model for different crack should be different. Therefore, using the recorded structural crack widths in Table 2 and the measured BGSs, a crack width prediction model was established for each of the four cracks. Similarly, the linear and quadratic prediction model were compared using the AICc. It was found that the linear model has smaller AICc values for all four cracks. Thus, the linear model was adopted, and the model parameters were estimated using the maximum likelihood method. To verify the accuracy of the obtained prediction models, the measured and predicted crack widths of the four cracks are compared. The comparison results are shown in Figure 26, illustrating that the prediction models can relatively accurately predict the structural crack widths (i.e., the largest relative prediction error is less than 20%).

Moreover, if the model coefficients of the four prediction models are compared, it will be found that these four prediction models are different as expected. It is different from the simulation result in Section 3.5 that the all cracks can share a same crack width prediction model. It may be due to the fact that in the simulation, the cracks were well separated apart such that the crack-induced strain change in the fiber is the result of only one crack; while, in the experiment there are many small cracks near the four major cracks. As a result, the small cracks also affect the strains in the fiber near these major cracks, leading to the change of the relationship between the crack width and the parameters $P_{g_{0}}$ and $P_{\Delta v_{B}}$. This experimental result suggests that to more accurately predict the structural crack width on the real structures, some modifications of the current model, accounting for the effects of adjacent multiple cracks, are needed.

## 5. Discussions About the Practical Application Issues of the Proposed Method

In this study, a new method was proposed to identify structural cracks, in which the BGS parameters (i.e., the peak Brillouin gain factor $g_{0}$ and Brillouin gain linewidth $\Delta v_{B}$) before and after the occurrence of structural cracks are utilized. Two parameters, $I_{g_{0}}$ and $I_{\Delta v_{B}}$, were constructed based on the above parameters to quantify the changes of $g_{0}\;$and $\Delta v_{B}$, respectively, due to structural cracks, which are then used to estimate structural crack location and width. However, it is well known that the development of structural cracks is a very slow process. Thus, continuous monitoring is not necessary, and periodical measurements are enough to obtain the development condition of structural cracks. During the two periodical measurements, the Brillouin demodulator is often used in another project, which is economical in practice. Then, a critical issue is raised whether or not the BGSs obtained at two measurement instances are comparable, particularly when some measurement conditions (e.g., power strength of CW light in Brillouin demodulator, cleanness of fiber connectors) are changed. Because during different measurements, the slightly change of measurement conditions are unavoidable. If the two BGS are not comparable under such a circumstance, the proposed method cannot be reliably applied in practice.

Actually, the BGS obtained from the Brillouin demodulator is related to the power strength of the CW light. The larger the power strength of the CW light, the larger the values of the BGS will be. However, if the fiber strain is not changed, the shape of the BGS will not change and the values of the BGS are simply changed by a factor, which will affect the peak Brillouin gain factor $g_{0}$ but not the Brillouin gain linewidth $\Delta v_{B}$. To alleviate the effects of changing power strength of the CW light, the following method can be adopted: instead of directly using the measured BGS, the normalized BGS, which equals the measured BGS divided by the average value of the BGS at all sweep frequency points, will be used to calculate the peak Brillouin gain factor $g_{0}$. It can be easily shown that the peak Brillouin gain factor $g_{0}$ obtained from the normalized BGS will not change with the change of the BGS via multiplying a constant. Therefore, through adopting the normalized BGS, the effects of the variation of power strength of the CW light on the identification results of $g_{0}$ can be removed.

## 6. Conclusions

Monitoring the initiation and development of structural cracks is of great importance to accurately access structural damage conditions and ensure the safe operation of structures. In this work, we presented a new crack identification method, which combines the DPP-BOTDA technology with the crack-induced irregular features of the BGS in the fiber. The DPP-BOTDA makes use of the differential Brillouin gain of two pump pulse lights with slightly different width to significantly improve the spatial resolution of strain sensing, which makes it possible to more clearly capture the large strain changes in the fiber near structural crack. The characteristics of the BGS in the fiber with the presence of structural crack were thoroughly investigated through numerical simulation. It was found that with the increase of structural crack, the peak Brillouin gain factor and the Brillouin gain linewidth are greatly reduced and increased respectively. Based on this discovery, a new crack indicator and a crack width prediction model were proposed, which was found to be effective in detect the occurrence of structural crack and predict the structural crack width by only using the measured BGS. Finally, an experiment was conducted on a simple supported RC beam, the results of which demonstrated that by using the BGS measured by the DPP-BOTDA, the proposed structural crack identification method successfully detects the structural cracks and relatively accurately predicts the crack widths.

Although the simulation and experimental results in this paper have proved the effectiveness of the proposed structural crack detection and structural crack width prediction methods, there is an important issue not fully solved. The simulation work in this paper showed that if there is only one crack and it is well separated from other cracks, the proposed crack width prediction model can very accurately predict the true structural crack width. However, in practice the structure may have several adjacent cracks, which deteriorates the accuracy of the prediction model. Therefore, how to account for the effects of multiple adjacent cracks on the structural crack width prediction will be a critical issue for the future research.

## Figures and Tables

**Table 1:** Comparison of standard deviation of prediction error and AICc value of two crack width prediction models.

Model Type	σ (mm)	AICc
Linear model	0.0027	−28.5
Quadratic model	0.0006	−31.5

**Table 2:** Crack width of tested beam under different load level (mm).

Crack No.	Load Level (kN)								
	40	80	120	160	200	240	260	280	290
1	0.06	0.1	0.13	0.14	0.16	0.17	0.23	0.27	0.21
2	0.02	0.05	0.08	0.10	0.12	0.14	0.15	0.18	0.13
3	0.04	0.07	0.1	0.11	0.15	0.16	0.18	0.2	0.16
4	0.02	0.04	0.04	0.05	0.06	0.05	0.06	0.06	0.07
5	0.03	0.07	0.08	0.12	0.14	0.16	0.16	0.53	0.82
6	0.03	0.07	0.11	0.14	0.15	0.18	0.22	0.17	0.82
7	0.02	0.05	0.06	0.07	0.05	0.05	0.05	0.07	0.09
8	0.03	0.06	0.07	0.10	0.10	0.13	0.12	0.11	0.11
9	0.02	0.05	0.08	0.09	0.13	0.18	0.20	0.25	0.24
10	0.02	0.05	0.07	0.08	0.07	0.10	0.10	0.08	0.11
11	0.02	0.06	0.07	0.08	0.11	0.13	0.13	0.15	0.13
12	0.03	0.07	0.09	0.12	0.13	0.16	0.18	0.21	0.18
13	0.02	0.05	0.05	0.07	0.08	0.07	0.1	0.07	0.1
14	0.02	0.05	0.07	0.08	0.1	0.13	0.14	0.16	0.17
15	0.01	0.03	0.07	0.08	0.08	0.09	0.11	0.13	0.17
16	0.03	0.07	0.07	0.10	0.10	0.10	0.13	0.15	0.13
17	0.01	0.02	0.07	0.07	0.05	0.05	0.07	0.08	0.05
18	0.02	0.05	0.08	0.12	0.11	0.14	0.16	0.21	0.18
